# Elucidating challenges and solutions in the maternal healthcare, identified by medical doctors in northern South Africa: a qualitative study

**DOI:** 10.11604/pamj.2020.36.376.18544

**Published:** 2020-08-31

**Authors:** Carl-Johan Valentin Olseén, Tebogo Mothiba, Linda Skaal, Stefan Rocco Hansson, Vanja Berggren

**Affiliations:** 1 Department of Health Sciences, Lund University, Södra Förstadsgatan 35, Malmö, Sweden,; 2 Department of Neuroscience, Care and Society, Karolinska Institutet, Alfred Nobels Allé 23, Huddinge, Sweden,; 3 Faculty of Health Sciences, Dean´s Office, University of Limpopo, Sovenga, South Africa,; 4 Department of Public Health, University of Limpopo, Sovenga, South Africa,; 5 Department of Clinical Sciences Lund, Lund University, Sölvegatan 19, Lund, Sweden,; 6 Obstetrics and Gynecology, Skåne University Hospital, Klinikgatan 12, Lund, Sweden

**Keywords:** Maternal healthcare, South Africa, sustainable development goals, challenges

## Abstract

**Introduction:**

as South Africa's maternal mortality ratio increased between 1990 and 2015, the country failed to reach the United Nations millennium development goal 5a. The maternal mortality ratio of Limpopo province is higher than the national average and previous studies report shortages of manpower and medical equipment in Limpopo province. The overall study aim was to elucidate views and experiences of medical doctors regarding maternal healthcare by identifying the challenges they experience and solutions they suggest.

**Methods:**

a qualitative interview-based study was performed with ten medical doctors as participants. Manifest content analysis was used to analyze the data.

**Results:**

the main findings were categorized as lack of material and human resources, feelings of experienced isolation and solution-focused expressions. The challenges identified included logistical issues, staffing issues, demographic characteristics of the patient population, poor interinstitutional communication and lack of support from the administration. The solutions included revision of resource allocation and improvement of the interinstitutional cooperation. For example, participants suggested that exchange programs between hospitals could be arranged, that the emergency medical service personnel could triage patients and that private practitioners could be contracted to work at public institutions.

**Conclusion:**

most identified challenges were related to a lack of resources. Based on their inside experience, the participants suggested several solutions. These firsthand accounts of the local medical doctors highlight the need for intervention and should be taken into account when it comes to improving the provincial healthcare and working toward achieving the healthcare-related sustainable development goals by 2030.

## Introduction

Several of the United Nations Sustainable Development Goals (SDGs) are intimately linked to women´s health [1], as were several of the United Nations Millennium Development Goals (MDGs). One example was MDG 5a, which was to reduce the maternal mortality ratio (MMR) by 75% [2]. The definition of MMR is the number of maternal deaths per 100,000 live births. The MMR of South Africa (SA) was estimated to be 138/100 000 by 2015, which is considered a moderate number. Between 1990 and 2015, the worldwide MMR was reduced by 44% [3]. During the same timeframe, the MMR of SA increased by 28%, indicating a failure to achieve MDG 5a [4]. The institutional MMR (iMMR) of SA is 116.9/100 000 as of 2017 [5]. It should be noted that studies performed on subnational levels report a declining MMR since the beginning of the 21^st^ century [6,7]. Some accredit this decline to higher coverage of Human Immunodeficiency Virus (HIV) testing and antiretroviral treatment [7,8], although it has been argued that the correlation between provincial MMR and HIV prevalence weak [9]. Limpopo is the northernmost province of SA. By 2011, its population was estimated to be slightly above 5,400,000, which accounted for 10% of the SA population [10]. Approximately 90% of the provincial population lives in rural areas and almost 50% are younger than 15 years old [11]. The province accounted for 7.2% of the national gross domestic product in 2010 [12]. According to the 2011 census, the unemployment rate of Limpopo was 38.9% [10] and the province had the lowest average household income of SA [13].

There is evidence of growing inequities in the maternal healthcare of SA. The nation-wide coverage of antenatal care (ANC) declined between 2008 and 2012 and the gap in access to these services between the poorest and the richest quartiles increased. Women under 20 years and those living in rural areas had markedly low ANC access [14], making the women of Limpopo province a vulnerable group. Indeed, the MMR of Limpopo is 130.2/100 000, which is 10.5% higher than the national average [5]. This study constitutes a part of a collaborative research program funded by the South African Medical Research Council (SAMRC) and the Swedish Research Council for Health, Working Life and Welfare (FORTE), focused on strengthening health systems for maternal care. As part of this program, workshops have been conducted to which Limpopo midwives were invited to share their experiences from and thoughts on maternal healthcare [15]. Together with other results, the present study might contribute to the production of a broad picture of the views of healthcare professionals. The aim of this study was to elucidate the views and experiences of medical doctors (MDs), regarding the maternal healthcare in Limpopo province by answering the questions: ‘which challenges regarding the maternal healthcare do the MDs of Limpopo experience?’ and ‘which solutions to these challenges do they suggest?´.

## Methods

**Study design:** the study aimed at elucidating experiences and views of MDs, using a qualitative explorative approach with an interview-based data collection [16,17].

**Sample:** the inclusion criteria were: a MD´s degree, experience in maternal healthcare and active duty in Limpopo province. The selection of the participants from the public sector was done with the assistance of the clinical managers at the maternity wards of two district hospitals, who facilitated a convenience sampling method. The participant working in the private sector was recruited via a conference. This participant had several years of experience in the public sector. No specialists or provincial hospital doctors were available for participation. The demographic data acquired from the participants are presented in Table 1. Participants received written and verbal information about the study and an interview consent form.

**Table 1:** demographic data of participants

Participant (n=10)	Institution	Years of practice	Age	Gender	First language
1	Private practice	26-30	50-59	Male	Sepedi
2	District hospital 1	0-5	30-39	Male	Tsonga
3	District hospital 1	0-5	20-29	Male	Venda
4	District hospital 1	11-15	50-59	Female	Sotho
5	District hospital 1	0-5	20-29	Female	Venda
6	District hospital 1	0-5	20-29	Male	Sepedi
7	District hospital 2	26-30	50-59	Male	English
8	District hospital 2	6-10	40-49	Female	Venda
9	District hospital 2	0-5	20-29	Male	Sepedi
10	District hospital 2	0-5	20-29	Female	Venda

**Data collection:** ten MDs were interviewed in May 2018. Nine interviews were conducted at the hospitals where the participants worked. One interview was conducted at a hotel. All were face-to-face semi-structured interviews and of high enough quality to be included in the study. An interview guide was used, consisting of 7 closed-ended questions related to the demographic data of the participants and 12 open-ended questions aimed to answer: ‘which challenges regarding the maternal healthcare do the MDs of Limpopo identify´ and ‘which solutions to these challenges do they suggest´. Verbatim transcriptions were produced from the recordings.

**Data analysis:** the transcripts were analyzed using qualitative manifest content analysis. As suggested by Graneheim and Lundman [18], the transcripts were first to read multiple times by the researchers to get an overview of the text. Next, the transcripts were read carefully and meaning units were identified. The meaning units were condensed, abstracted and labeled with codes. The codes were sorted into seven sub-categories, which in turn were sorted into three main categories. Analyst triangulation and an inquiry audit were done with the assistance of an external researcher.

**Ethical considerations:** it is important to guarantee the confidentiality of the participants of the study. No information presented can be tracked down to a single participant. Participants were informed that they had the right to withdraw from the study at any time without explanation, they were also given the contact information of the principal investigator in case they had further questions or wished to receive a copy of the interview transcript. The investigation conforms with the principles of the Declaration of Helsinki [19], and is covered by the ethical clearance received for the whole collaborative research program from the ethical board at the University of Limpopo (TREC/117/2017: IR).

## Results

Three main categories emerged from the analysis of the transcripts: lack of material and human resources, feelings of experienced isolation and solution-focused expressions. These main categories have 7 sub-categories, as presented in Table 2.

**Table 2:** the main categories and their respective sub-categories

Main category	Subcategory
Lack of material and human resources	Logistical issues
	Staffing issues
	Demographic characteristics of the patient population
Feelings of experienced isolation	Poor interinstitutional communication
	Lack of support from the administration
Solution-focused expressions	Revision of resource allocation
	Improved interinstitutional cooperation

***Lack of material and human resources*:** all participants mentioned the lack of material and human resources as the main challenges for the provision of quality maternal healthcare.

**Logistical issues:** the participants perceived that the hospitals did not receive the logistical support they needed to function at an optimal level. The lack of equipment was considered a challenge by all participants employed at public hospitals. They reported that the hospitals lacked both clinical and laboratory equipment, which made it difficult to diagnose and monitor patients and to perform different tests such as blood gas analysis. The participants also experienced that the available equipment often was malfunctioning. *“But if they say they want to reduce maternal death and we don´t even have a basic BP cuff, so how do you expect the perinatal death to go down if you can´t even take the BP?”*Moreover, several participants perceived that the facilities were substandard. They experienced that the hospitals were not big enough to accommodate the patients and that dysfunctional facilities forced them to transport patients in ways they perceived as dehumanizing. The limited capacities of the hospitals resulted in patients being discharged prematurely to make space. *“We don´t have beds, the hospital itself can´t accommodate patients. In each cubicle, we´re supposed to have only two patients, but you´ll find that we have six. Two on the floor, and two on every single bed”*.The poor access to drugs was also mentioned as a challenge by several participants employed at public hospitals. The lack of certain medications forced the MDs to provide inferior treatments or refer patients to the provincial hospital for adequate treatment. Lastly, the inefficiency of the Emergency Medical Services (EMS) was raised as a major challenge by nearly all participants. They described the transportation of patients as slow and often delayed and mentioned a lack of ambulance vehicles as the main reason for this. Furthermore, it was mentioned was that the EMS personnel were not authorized to triage their patients, which according to some participants led to the services being overwhelmed by patients who did not need to use them. *“They can even take the whole 12 hours without coming. There was a point where I had to use my own car to transport a dying patient to Polokwane because they didn´t come. That´s how bad it is. It´s bad”*.

***Staffing issues*:** the characteristics of the staff within the public sector were regarded as the main challenge for maternal healthcare. All participants emphasized the lack of manpower. They experienced a lack of nurses, MDs, EMS personnel and laboratory technicians. *“And the other challenge that I encountered is staffing. I´d say both from the nursing and the doctors´ side. Usually what I would encounter on a daily basis is that you´d find that during a clinic day, you have a lot of patients coming in and they all require vital signs, they all require a nurse to actually review them. But you find that all the cubicles in labor ward are full of patients in labor and you don´t have nurses, enough staffing, to actually give us attention during the clinic”*.The lack of laboratory technicians made it difficult to get test results at certain hours. *“If you try to access the lab, if we want for example urgent blood, you´ll find that these people, the lab technicians, are mostly not around. You´ll call them and they have to travel all the way to come and do those blood. Actually, we don´t have one on a call like each and every day”*.Procedures requiring more than one MD left other wards unmanned. The lack of manpower also left the MDs feeling obliged to work extra hours for which payment was uncertain. *“For now most of us have already done 72 hours and it's still in the middle of the month and if you do more than that nobody pays you. They say: ‘you can do extra hours, we'll pay you´ but nobody does. There is no document that we sign that says we'll get paid for those extra hours. Ideally, we're supposed to work from 72 to 80 hours, they pay up to 72 hours. The rest nobody covers”*.

The lack of competence among the staff was mentioned as another challenge. They reported that there were no doctors available at the peripheral clinics, that the nurses at the district hospitals did not always have sufficient training, that the junior doctors by far outnumbered the senior doctors at the district hospitals and that no specialist doctors were available at the district hospitals. *“It´s a rural area and sometimes we don´t have a lot of resources and even the staff, we have a lot of junior doctors, the senior doctors are not there. Basically, in short, that´s our major problems. In any environment we need senior doctors to be there to support the junior doctors because they are still learning some of the skills”*.These staffing issues were perceived to pose unnecessary risks for patients and increase the number of referrals made from the peripheral clinics to the district hospitals and from the district hospitals to the provincial hospital. *“They´re supposed to be sending us high-risk patients. So those are the patients who have hypertension, diabetes, previous caesarian section times one, or previous fetal death, previous miscarriages, advanced maternal age and so forth. So those are the patients a midwife can´t manage without a doctor. But then, because of the workload, you´ll find that they´re just sending us anything, even low-risk patients we end up managing this side, which makes our workload very high. We´re supposed to be seen only selected patients but it's not happening”*.

***Demographic characteristics of the patient population:*** the demographic characteristics of the patient population were considered a challenge by some of the participants. The participant working within the private sector addressed the fact that not everyone who requested his services could afford it due to the low socioeconomic status of the patient population. Sometimes he had to rely on the patients paying later since their funds were exhausted. Participants from the public sector informed us that some patients were not able to afford the drugs they were prescribed.

**Feelings of experienced isolation:** the rurally placed MDs expressed feelings of being cut-off from the provincial hospital, the neighboring district hospitals and the decision-making institutions, leaving them with a feeling of isolation.

***Poor interinstitutional communication*:** most of the participants considered the communication between the different hospitals, clinics or with the EMS personnel to be the main challenge. The doctors from district hospitals reported problems getting in touch with specialists for a second opinion or referrals. *“Yes, we´re having a problem with that. Even now I have an emergency, I have to refer that patient and I´ve been on the phone for more than an hour trying to call them, no answer”*.They also experienced a lack of understanding from the provincial hospital doctors regarding the limitations of the district hospitals, resulting in patients being refused for referral although further treatment was impossible at the district hospitals. One participant stated that the referral requirements were not standardized. *“There are some doctors who would deny patients based on the category that says that they only take from 1.2 when we know it´s 1000 grams. So we surely have those types of challenges. The transferring policy is not really standardized”*.The doctor from the private sector emphasized the lack of vertical integration as a problem, as he did not receive any feedback information about referred patients and that there was no cooperation between the sectors regarding laboratory testing and test results. *“Now, unfortunately, because of this lack of watching together between the public and the private sector, it tends to pose a problem in terms of proper maternal healthcare between the private sector and the public sector”*.

***Lack of support from the administration*:** some of the participants from the public sector mentioned the lack of support from the provincial or the governmental administration as a challenge. They reported that they were not provided the necessary resources, even though it was promised by decision-making organs. The also perceived that there was a lack of understanding of the capabilities of the district hospitals among the provincial and governmental administrative staff. One participant argued that hospital management was unmotivated to perform its best to ensure well-functioning healthcare. *“I´ve been here for 4 years now when I came here apparently they were saying that they were going to expand the hospital because they know that it´s small and we have a high patient load. It´s been four years now; the hospital is still the same. We have so many patients, we have patients who sleep on the floors, nobody cares”*.

### Solution-focused expressions

***Revision of resource allocation*:** most of the participants expressed that a solution to many of their challenges would be a revision of the resource allocation to allow an increased hospital and healthcare funding, which could be spent on equipment, staffing and facility improvement. A desire for more ambulance vehicles to be bought was also expressed. Some of the participants suggested that there could be ambulance vehicles dedicated to maternity cases. One participant suggested that there could be an ambulance stationed at the hospital to reduce transportation delays. *“And the other issue, I think I´ve raised this, is the issue of EMS transportation. I think that we can improve by actually having on-site at least one emergency vehicle on site for obstetric and also other emergency patients in the hospital. So that whenever there is an emergency that needs very urgent transportation, they will be able to transport the patient in time”*.

***Improved interinstitutional cooperation*:** the participant from the private sector suggested that when he referred a patient to a public hospital, he should be able to take samples and pre-order necessary tests to avoid delays. It was also suggested, to cope with the problem of lacking manpower, that private practitioner could be allowed to be contracted to work publicly. Furthermore, it was suggested that if there was better communication between district hospitals and the EMS personnel, emergency patients could be directed to hospitals currently able to accommodate them, in order to lessen the burden of high patient volume. One participant suggested that the EMS personnel should be authorized to do triaging, which would help increasing ambulance availability. *“I just think that the rules must be revised because some of them are really, you know, good and qualified EMS personnel, but they can't even triage patients at home and decide like ‘we can´t take a headache´. Because it´s so painful when you see a headache or someone with back pain for two weeks come in with an EMS, then you see that patient for 30 minutes or less, you give some voltaren or whatever, then the patient goes home. Where else there was someone really dying who could have used that”*.Some participants from the public sector suggested that in order to improve the competition among the district hospital MDs, the capacity building activities between the provincial and the district hospital should be increased and improved. It was suggested that the district hospital doctors could be allowed to visit the provincial hospitals to interact with specialists during ward rounds, meetings or conferences. Some participants suggested that specialists could come to district hospitals and perform teaching rounds. Some junior doctors also expressed a wish to be notified when teaching events were being arranged at the provincial hospital. *“I´ve never received an e-mail or something, we don´t actually even know where to go to look for conferences. For sure there is always conferences happening somewhere around in Polokwane. If they can leak that information to us; ‘there’s a conference here, please attend´. Even if we had to pay, we´d go”*.

## Discussion

The principal findings from this study are that the interviewed doctors perceived the Limpopo hospitals, clinics and EMS as lacking the core equipment and manpower needed to provide well-functioning maternal healthcare. Most of them thought that with more of these resources, many of the problems could be solved. A study conducted at the University of Limpopo in 2015 estimated that Limpopo housed 887 MDs (community service doctors excluded), making the density of MDs 16.4 per 100,000 inhabitants. Of these MDs, 776 were medical officers, of which 43% were employed in the Capricorn District and the majority at the provincial hospital. Only 99 of the 887 MDs were specialists and 78% of those were employed at the provincial hospital. Only 2% of the specialists were employed at district hospitals. The provincial density of specialized MDs was 1.8 per 100,000 inhabitants. The study confirms that there is a general shortage of doctors, particularly specialists, in Limpopo [20], which correlates with earlier investigations stating there is a national shortage of doctors in SA [21,22]. In 2014, Ntuli and Ogunbanjo showed that Limpopo referral hospitals also lack midwives [23]. The present study shows that the active MDs experience this lack of manpower, competence and material as a challenge. The ineffective EMS was also perceived as a challenge for Limpopo healthcare, mainly due to lack of vehicles and poor communication. These observations correlate with the results from a systematic review by Kironji *et al*. published in 2018, in which they identified similar limitations for the Out of Hospital Emergency Care (OHEC) in low-middle income countries. Among their findings were poor communication, poor access to ambulance vehicles, lack of equipment and lack of skills among the EMS personnel which is in accordance with what the participants of this study described. They concluded that the focus of improvement should be on means of transportation, training and patient access to the OHEC system [24].

A qualitative study interviewing nurses at a district hospital in SA also showed that there was a critical shortage of equipment and that the available equipment was of low quality. The said study concluded that functional equipment needs to be provided in order to increase the quality of healthcare [25]. One of the most emphasized findings of the present study was the perceived challenge of hospitals lacking equipment. The doctors suggested that in order to minimize this challenge, they need to be provided with equipment adequate in both quality and quantity. Examples of material the participants expressed a need for were ultrasound machines, pulse oximeters, blood pressure cuffs, endotracheal tubes and various drugs. Many participants experienced that the communication between the district hospitals and the provincial hospital is dysfunctional. It is likely the communication problems originate in the provincial hospital doctors facing much of the same logistical challenges as the district hospital doctors. An investigation at the Limpopo provincial hospital suggests that the hospitals high iMMR might originate in low-quality care at the district hospitals due to lack of competence and poor referral systems. The investigation also states that the provincial hospital is understaffed and lacks equipment [26]. This studies´ interviews were performed in different environments, most of them in clinical settings. For further studies, more relaxed interview environments would be preferable to increase credibility and dependability.

As the views of the MDs of Limpopo regarding the maternal healthcare of the province is a previously much-unexplored issue, there are at present no internationally published studies available to compare strengths and weaknesses with. For the same reason, there are no previous results to compare with, although several studies, as presented above, show results which correlate with the issues perceived as problematic for the healthcare by the participants of this study. The meaning of this study is emphasized by the lack of previous research conducted on the matter. In summary, the different studies reflecting the views and experiences of the nurses and midwives of Limpopo, together with results from the current study, may help to give an overall view of the problems identified by healthcare professionals relating to healthcare in general and maternal healthcare specifically. Future studies should further explore the experiences and views of the healthcare professional at the provincial hospital level, as well as on a community-based level, also including a specialist perspective. Moreover, further research of quantitative design is needed to provide possibilities to generalize. Although issues related to some of the results of this study have been previously raised, the firsthand accounts of the MDs of the province helped to highlight the need for intervention and should be taken into account when it comes to improving the provincial healthcare and working toward achieving the healthcare-related SDGs by 2030.

## Conclusion

The present study shows that the MDs in Limpopo identify a variety of clinical challenges regarding the maternal healthcare of the province. The challenges that were most emphasized were related to a lack of material, manpower and skills. The ineffective emergency transportation system and poor interinstitutional communication were also raised as major challenges. The MDs suggested that a revision of the resource allocation would solve many of their logistical problems. The participants also expressed a need for improved interinstitutional cooperation in terms of capacity building and patient assessment.

### What is known about this topic

- The MMR of Limpopo is higher than the South African national average;
- The healthcare of Limpopo is facing problems such as lack of material and manpower;
- The women of Limpopo are a vulnerable population in terms of access to maternal healthcare.

### What this study adds

- The MDs of Limpopo identify a shortage of material and manpower as the major challenges for the maternal healthcare of the province;
- The MDs of Limpopo identify the lack of functional interinstitutional communication as a major challenge for the maternal healthcare of the province;
- The MDs of Limpopo express a need for increased cooperation between different healthcare institutions.
